# Quantification of breast cancer risk based on the UK five-point classification system

**DOI:** 10.1186/bcr2958

**Published:** 2011-11-04

**Authors:** D Tzias, S George, L Wilkinson, R Mehta

**Affiliations:** 1 St George's Hospital, London, UK

## Introduction

The UK five-point classification for radiological assessment of mammograms and ultrasound scans broadly enables evaluation of cancer risk [1], but no specific probabilities are incorporated into this system. By comparison, the widely accepted BI-RADS scoring system does include percentage cancer risk for each category [2]. Our study sought to investigate the cancer probability for each of the five radiological grades in the UK scoring system amongst a large group of mammograms.

## Methods

The reports of 3,149 mammograms performed over a 10-month period within the symptomatic breast service at St George's Hospital, London were analysed. The corresponding histopathology reports were collected for identification of malignant cases. Percentage cancer risk was calculated for each category within the UK five-point classification system.

## Results

The pathology reports corresponding to each of the 3,149 mammograms revealed 78 cases of malignancy. Data analysis gave the following cancer probabilities for each category: M1, 0.3%; M2, 0.6%; M3, 13.5%; M4, 63.6%; and M5, 83.0%.

## Conclusion

We propose that calculation of cancer risk for each category within the UK five-point scoring system is a valuable parameter. It enables accurate performance monitoring within a breast unit as well as comparison with national/international standards.
